# Giant villous adenoma of rectum- what is the malignant potential and what is the optimal treatment? A case and review of literature

**DOI:** 10.1186/s12957-019-1650-4

**Published:** 2019-06-25

**Authors:** Lovenish Bains, Pawanindra Lal, Anubhav Vindal, Meeta Singh

**Affiliations:** 1 Department of Surgery, Maulana Azad Medical College, New Delhi, India; 2 Department of Pathology, Maulana Azad Medical College, New Delhi, India

**Keywords:** Villous, Adenoma, Giant, Rectal, Malignant, Colorectal carcinoma, Laparoscopic, Colo-anal

## Abstract

**Introduction:**

Villous adenomas are dubiously benign lesions, which are difficult to interpret because of their malignant potential. Distal villous adenomas present with bleeding or mucus discharge. Giant adenomas are not amenable for endoscopic or transanal resection. Only few isolated cases have been reported by laparoscopic resection. We present our case of a circumferential giant villous adenoma of the rectum managed successfully by laparoscopic ultra-low anterior resection with colo-anal anastomosis with a review of literature in regard to their malignant potential.

**Case report:**

A 62-year-old lady presented with complaints of painless bleeding per rectum and a fleshy mass protruding from the anal canal which on digital rectal examination appeared a large soft velvety flat mass with mucus discharge. Colonoscopy showed circumferential irregular, friable, edematous mucosa in rectum extending for 15 cm. Computed tomography showed a large heterogeneously enhancing polypoid mass lesion in the rectal wall involving the entire rectum. The patient underwent laparoscopic low anterior resection with colo-anal anastomosis and protecting loop ileostomy. Histopathological examination of the resected specimen revealed villous adenoma of the rectum with moderate to severe dysplasia.

**Discussion:**

Villous adenomas are sessile growths lined by dysplastic glandular epithelium, whose risk of malignancy is especially high up to 50% when greater than 2 cm in size. Large size, villous content, and distal location are all associated with severe dysplasia in colorectal adenomas. Large villous rectal tumors, particularly of circumferential type pose a great challenge for endoscopic or transanal removal. Henceforth, open or laparoscopic surgery is required for these cases.

**Conclusion:**

Giant rectal villous polyps are usually unresectable by endoscopic methods or transanal endoscopic microsurgery and are associated with a high rate of unsuspected cancer which requires a formal radical oncologic resection. As per current data, the combined risk of dysplasia/malignancy is about 83% with 50% risk of dysplasia and frank malignancy in 33% of cases of giant rectal villous adenomas of more than 8 cm in size. Laparoscopic colorectal resection is safe and effective.

## Introduction

The prevalence of adenomatous polyps of the colon and rectum was reported in approximately 25% of the population aged over 50 years [1]. A recent meta-analysis puts the pooled prevalence in average-risk individuals of adenomas, colorectal cancer, non-advanced adenomas, and advanced adenomas at 30.2%, 0.3%, 17.7%, and 5.7%, respectively [2]. The larger villous adenomas with severe dysplasia are mostly concentrated in the distal colon (left colon and rectum), in particular in the descending-sigmoid part [3]. As giant adenomas are difficult for endoscopic removal and malignant potential is not known, laparoscopic colectomy offers safe and effective management of these lesions with the benefits of accelerated postoperative recovery [4]. We present a case of a circumferential giant villous adenoma of the rectum managed successfully by laparoscopic ultra-low anterior resection with colo-anal anastomosis and review of literature in terms of malignant potential and optimal treatment of such tumors.

## Case summary

**Fig. 1:**
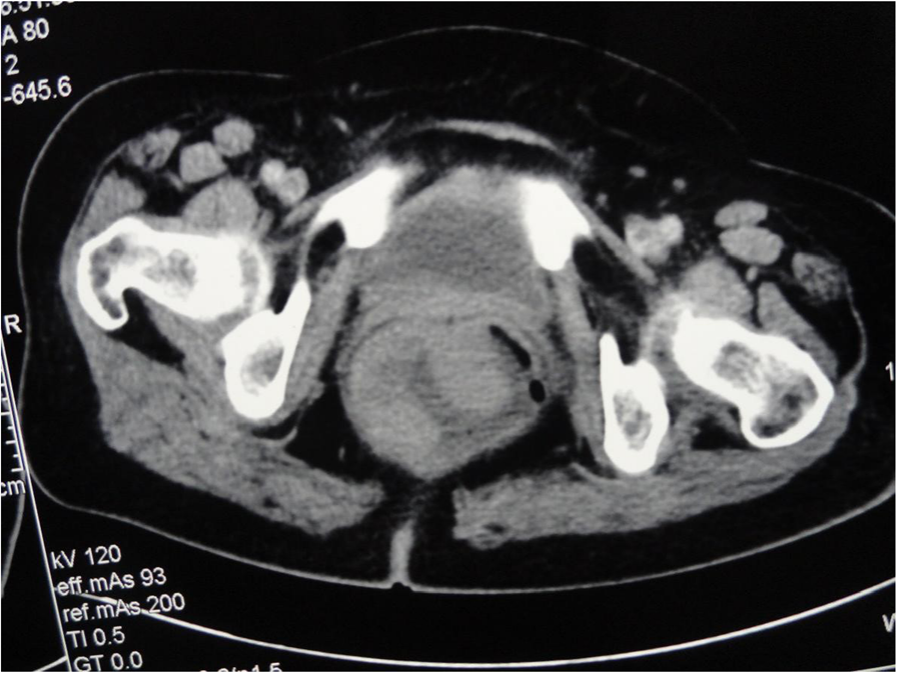
CECT (transverse section) showing the tumor almost involving entire circumference of rectum

**Fig. 2:**
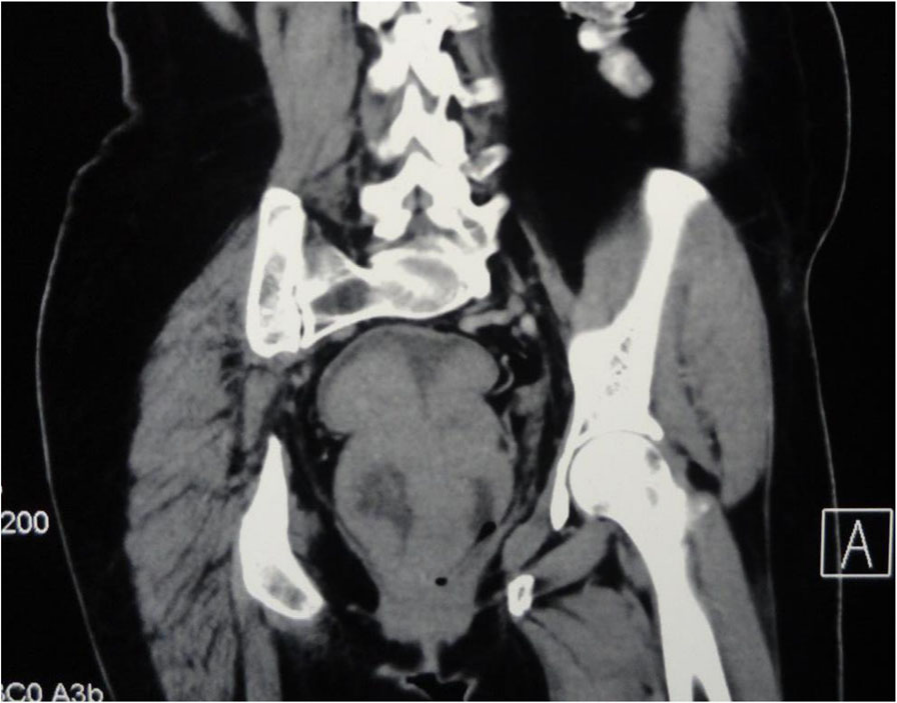
CECT (coronal section) showing the tumour length

A 62-year-old lady presented to our outpatient department with complaints of painless bleeding per rectum from 8 months and a fleshy mass protruding from the anal canal from 3 months. The mass was reducible on manual palpation and associated with profuse mucus discharge. The hydration status of the patient was adequate, she had mild pallor and abdominal examination was essentially normal. On digital rectal examination, a soft velvety flat mass was appreciated about 3 cm from anal verge extending from 9 o’ clock to 5 o’ clock along with mucus discharge. The upper extent of the lesion could not be reached. The laboratory investigations revealed hemoglobin 8.3 g%, normal electrolytes with no other abnormalities; carcinoembryonic antigen (CEA) was also within normal range. Initial biopsy revealed villous adenoma with focal moderate dysplasia. A colonoscopy was done which showed irregular, friable, edematous mucosa in rectum extending for 15 cm nearly circumferentially with no other lesions in the entire colon. Contrast-enhanced computed tomography showed a large heterogeneously enhancing polypoid mass lesion in the rectal wall (max thickness 2.5 cm) involving the entire rectum (Figs. 1 and 2). Surrounding fat planes were normal and there were no enlarged lymph nodes.

In view of the large size of the lesion with associated moderate dysplasia, a decision was made to perform a laparoscopic low anterior resection. The rectum and sigmoid colon were mobilized with high ligation of the inferior mesenteric artery, total mesorectal excision with circumferential radial margin up to the dentate line and divided beyond the lesion. Colo-anal anastomosis was performed using PROXIMATE® ILS curved intraluminal stapler (25 mm) (Ethicon, Johnson, and Johnson, Cincinnati, OH, USA) and protected by a proximal loop ileostomy. The patient recovered well and was discharged on the fourth post-op day. At the time of discharge, digital rectal examination revealed a preserved anal sphincter tone and no troublesome mucus discharge.

**Fig. 3:**
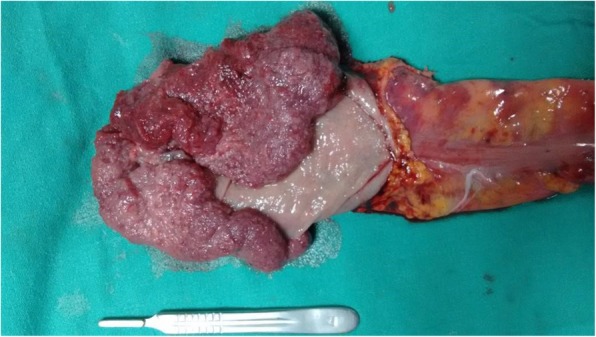
Resected specimen (everted) showing the villous tumor, circumferential

**Fig. 4:**
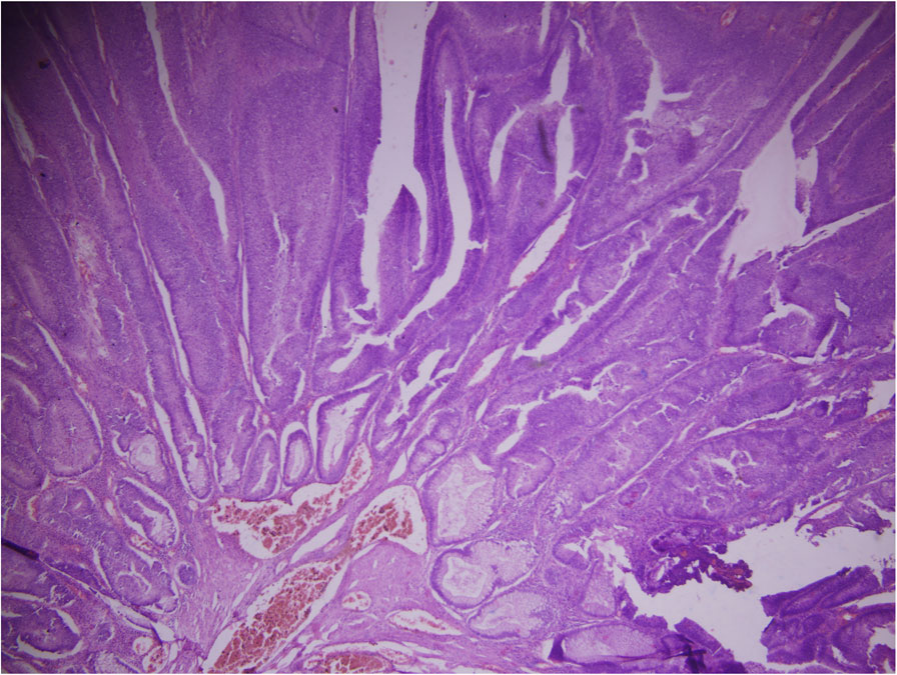
Histopathology showing villous architecture, × 40, H&E

**Fig. 5:**
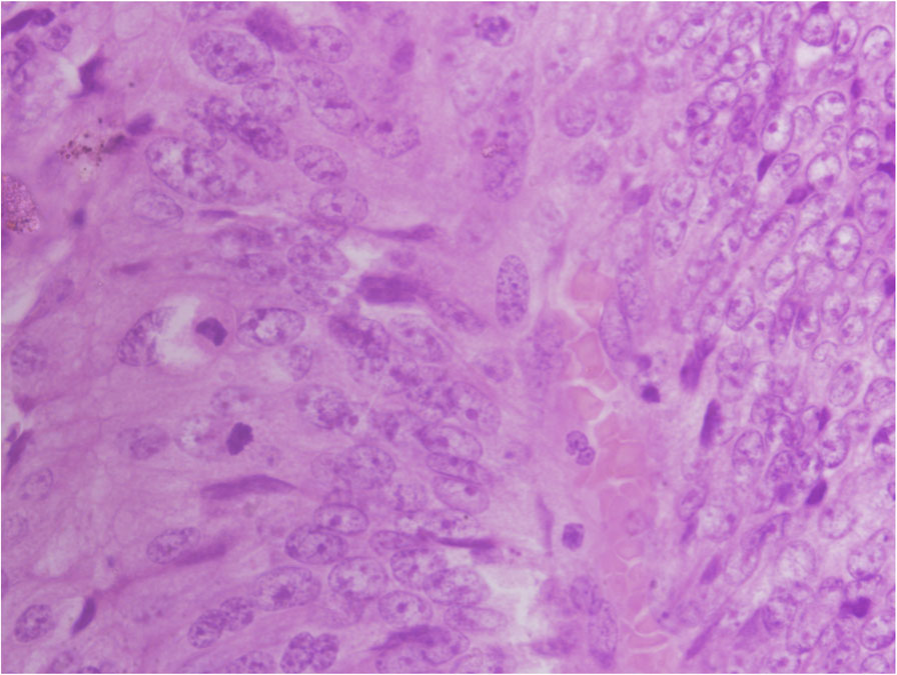
Dysplasia as atypical mitotic figures, × 600, H&E

On examination of the specimen, the lesion was seen to measure 16 × 12 cm, involving almost whole circumference with a 20 cm proximal and 1 cm distal margin (Fig. 3). Histopathological examination of the resected specimen revealed villous adenoma of the rectum with moderate to severe dysplasia (Figs. 4 and 5). Both the resected ends were free of tumor. Fourteen lymph nodes were isolated, all of which showed reactive changes. The patient underwent restoration of bowel continuity after 5 months and is healthy up to 1 year of follow-up.

## Discussion

The natural history of colorectal carcinomas has been extensively studied in correlation with the underlying accumulation of genetic alterations as understood by the adenoma-carcinoma sequence. Adenomas are precursor lesions defined by the presence of intraepithelial neoplasia, characterized by varying degrees of nuclear stratification and loss of polarity. Polyps develop as mucosal excrescence as a consequence of accelerated crypt fission resulting from APC gene mutation [5].

The ‘villous tumor’ of the rectosigmoid was first described by Que’nu and Landel in 1899. They described very large broad-based rectal tumors associated with secretory diarrhea [6]. The age-related prevalence of colorectal adenoma is 21–28% in 50–59 years old, increasing to 40–45% in 60–69 years old and rising to 53–58% in people over the age of 70 [7]. Histologically, polyps are classified as neoplastic (adenomas) or non-neoplastic. Non-neoplastic polyps have no malignant potential and include hyperplastic polyps, hamartomas, lymphoid aggregates, and inflammatory polyps. Neoplastic polyps or adenomas have malignant potential and are classified according to the World Health Organization as tubular, tubulovillous, or villous adenomas, depending on the presence and volume of villous tissue [8]. Most of them (70% to 85%) are classified as tubular (< 25% of villous tissue), 10–25% are tubulovillous (25–75% of villous tissue), and 5% are villous adenomas (75–100% of villous tissue) [8]. Villous adenomas are classically sessile with a velvety or hairy surface and microscopically leaf-like projections lined by dysplastic glandular epithelium. Villous architecture is defined arbitrarily by the length of the glands exceeding twice the thickness of normal colorectal mucosa [8, 9].

Giant polyps are usually defined as more than 3 cm on endoscopy [10, 11]; however, limited literature is available about optimal management of giant villous adenomas more than 10 cm [12, 13]. Our case is 16 × 12 cm which was successfully managed with laparoscopic technique.

The risk of malignant degeneration is related to both the size and type of polyp [2, 9, 10]. Tubular adenomas are associated with malignancy in only 5% of cases, whereas villous adenomas may harbor cancer in up to 40% [2, 10]. Tubulovillous adenomas are at intermediate risk (22%). There is less than a 5% incidence of carcinoma in an adenomatous polyp less than 1 cm in size, whereas there is a 50% chance that a villous adenoma greater than 2 cm in size will contain cancer [10, 11]. The risk of malignancy is especially high when adenomas are large (> 10 mm) and multiple, with a villous pathology [2, 3, 9]. Not all neoplastic polyps evolve to cancer but most colorectal cancers originate as a polyp. This fact forms the basis for secondary prevention strategies to eliminate colorectal cancer by targeting the neoplastic polyps for removal before malignancy develops [2, 3].

A recent search on PubMed for keywords ‘giant rectal villous adenoma’ and ‘giant rectal villous tumor’ from 2005 to 2018 yielded 33 and 31 results respectively. The keyword ‘malignant potential of rectal villous adenoma’ yielded 24 results A total of 25 giant villous tumors (including our case) with size range 5–31 cm were reviewed which showed carcinoma (including invasive) in 8, high-grade dysplasia in 6, low- to moderate-grade dysplasia in 6 whereas rest negative for malignancy (Table 1). It puts the risk of dysplasia to about 50 % and malignancy in 33 % of cases of giant rectal villous adenomas. This analysis suggests that endoscopically unresectable polyps or giant polyps of villous type are best treated by radical oncologic resection.

**Table 1.** Giant villous tumor of rectum in recent literature with size and malignant potential

S no.	Author	Size	Histopathology
1.	Rickenbacher A, Bauerfeind P et al. [12]	1. 15 cm 2. – 3. 8 cm	-Low-grade dysplasia -High-grade dysplasia -Giant tubulovillous adenoma with a small focus of invasive carcinoma
2.	Durán-Martínez M, Medina-Fernández FJ et al. [13]	10 × 8 × 4 cm	Giant villous adenoma high-grade dysplasia
3.	van der Pool AEM, de Graaf EJR et al. [14]	1. 12 cm 2. 24 cm^2^ 3. 5–14 cm or 99 cm^2^	-Villous adenoma -Villous adenoma with high-grade dysplasia and intramucosal carcinoma -Villous adenoma with low-grade dysplasia
4.	Agnes A, Novelli D et al. [15]	9 cm	Villous adenoma with areas of intramucosal adenocarcinoma and high-grade dysplasia
5.	Challis BG, Lim CT et al. [16]	10 cm	Tubulovillous adenoma exhibiting moderate to low-grade dysplasia
6.	Okano M, Okuyama M et al. [17]	10 cm	
7.	Mois EI, Graur F et al. [18]	14 cm	Low-grade dysplasia with focal high-grade dysplasia
8.	Nakhla SG, Murakami TT et al. [19]	17 cm	Large rectal villous adenoma coexistent with a poorly differentiated neuroendocrine tumor of the rectum
9.	Kure K, Kawai M et al. [20]	12.7 × 11.5 cm	Mostly tubulovillous adenoma, but partially moderately differentiated adenocarcinoma.
10.	Das P, Vijay MK et al. [21]	8.5 × 6 × 4 cm	Giant villous adenoma with low-grade dysplasia
11.	Ohtsuka M, Hata T et al. [22]		Concurrent adenocarcinoma
12.	Roriz-Silva R, Andrade AA et al. [23]	14 cm	Villous adenoma with low-grade atypia
13.	Aboul Hosn M, Abdel-Hafiez N et al. [24]	12 cm	No evidence of invasive carcinoma
14.	Barendse RM, van den Brandt S et al. [25]	9 cm	Villous adenoma with focal high-grade dysplasia
15.	Choi WH, Ryuk J et al. [26]	25 cm × 12 cm	Well-differentiated adenocarcinomas arising in villotubular adenomas
16.	Tuţă LA, Boşoteanu M et al. [27]	12 × 10 cm	Well-differentiated adenocarcinoma arising within a villous adenoma.
17.	Dagan A and Reissman P [28].	31 cm	Low- to high-grade dysplasia
18.	Cubuk R, Tasali N et al. [29]	17 × 9 cm	Villous adenoma without high-grade dysplasia
19.	Koning GG, Rensma PL et al. [30]	15 × 8	Low-grade dysplasia
20.	Nagri S, Eskaros S et al. [31]	8 × 5 × 4 cm	Giant villous adenoma with high-grade dysplasia
21.	Bains L, Lal P et al. (current case)	16 × 12 cm	Giant villous adenoma of the rectum with moderate to severe dysplasia

In a study that analyzed 7590 adenomatous polyps to determine risk factors for high-grade dysplasia or invasion, the size was the strongest predictor. The percent of adenomas with high-grade dysplasia or invasive cancer based on the size of the polyp was as follows: <5 mm, 3.4%; 5–10 mm, 13.5%; and > 10 mm, 38.5% [11]. Large size, villous content and distal location are all associated with severe dysplasia in colorectal adenomas [3, 8–10].

Most patients with adenomas are asymptomatic, especially when their neoplasm is identified by screening or surveillance [2, 7, 9, 10]. Hematochezia and anemia are common presenting features due to bleeding from the tumor. Rectosigmoid lesions can present with protrusion of mass or tenesmus. Other symptoms include fever, malaise, weight loss, and abdominal pain. Villous rectal tumors may reach a large size, and look like a “rug” involving the entire rectum, without degenerating into malignant disease [6]. An important fact is that the giant villous adenomas may excrete large quantities of mucus and potassium, which can produce mucus diarrhea and electrolytic alterations. McKittrick-Wheelock syndrome, which is a disorder characterized by fluid and electrolyte depletion, is caused by a secretory colorectal tumor [32].

Colonoscopy is the procedure of choice for diagnosing colorectal polyps as it is the most accurate method for detecting polyps of all sizes, and it allows biopsy of lesions and resection of most polyps [2, 33, 34]. Endoscopic polypectomy is the mainstay of polyp management because the majority of lesions are protuberant. Polyp size, position, and access can make this very taxing and a great challenge for endoscopic or transanal endoscopic microsurgery (TEMS) removal. Large villous tumors of the low and mid rectum can be treated by per-anal resection with recurrence rates equivalent to transanal endoscopic microsurgery; however, the mean length of the tumor was 5.2 cm in this series [35]. TEMS can be employed in lesions up to 6 cm in carefully selected patients but owing to giant size, location, circumferential, and diffuse villous lesion, it has not been indicated for such lesions. Such big lesions may approach the dentate line and pose an increased risk of perforation with serious complications. Another unfavorable point of endoscopic resection of circumferential rug like mucosa is the development of stricture [36–39].

Carditello et al. treated 104 villous tumors of the rectum with a mean size of 3 cm surgically by local or wide excision. The malignant potential of the tumors was 30%, including 10% invasive malignancy and recurrence rate was 24 after a mean follow up of 6.5 years [40]. A recent study found that the incidence of cancer in patients undergoing colectomy for an irretrievable polyp is 17.7% [39]. Open or laparoscopic colorectal resection is the procedure of choice for lesions not eligible for endoscopic resection and for large sessile villous tumors [4, 23, 26, 39]. It has been demonstrated a mortality rate of 0.3% with an anastomotic leak rate of 1.4% [4, 11, 39]. Complete excision is warranted for rectal villous adenomas, as biopsies were accurate only 50% of the time, and 1 in 8 patients had unsuspected cancer found after excision [35, 37, 41].

## Conclusion

Giant villous adenoma is a high-grade dysplastic lesion with conversion rate from adenoma to carcinoma approaching 17–33%. Troublesome mucus discharge and bleeding may result in severe hemodynamic alterations. As per current data, the combined risk of dysplasia/malignancy is about 83% with 50% risk of dysplasia and frank malignancy in 33% of cases of giant rectal villous adenomas of more than 8 cm in size. Giant rectal villous polyps are usually unresectable by endoscopic methods or transanal endoscopic microsurgery and are associated with a high rate of unsuspected cancer which requires a formal radical oncologic resection. Laparoscopic colorectal resection is safe and effective.

## Data Availability

The data supporting the conclusions of this article are included in the article.
